# Outcome of patients with osteosarcoma over 40 years of age: Is angiogenesis a marker of survival?

**DOI:** 10.1186/1477-7800-3-7

**Published:** 2006-03-21

**Authors:** Eugene TH Ek, Joseline Ojaimi, Yasuyuki Kitagawa, Peter FM Choong

**Affiliations:** 1 Department of Orthopaedics, University of Melbourne, St. Vincent's Hospital, Melbourne, Australia; 2 Division of Surgical Oncology, Peter MacCallum Cancer Institute, Melbourne, Australia

## Abstract

**Background:**

Osteosarcoma predominantly afflicts young people in their second and third decades of life. When osteosarcoma arises in patients older than 40 years, the prognosis is usually poorer compared to their younger counterparts. Although the clinical, histopathologic features and prognostic indicators are well defined for young patients, much less is known about affected adults. The purpose of this study is to describe our institution's experience with the management of osteosarcoma in patients greater than 40 years and also evaluate, by immunohistochemical analysis, the prognostic significance of microvessel density, as a marker of intratumoural angiogenesis.

**Methods:**

A retrospective clinicopathological analysis was performed on 11 patients over the age of 40 years that were treated at our institution between 1996 and 2004. Archival pre-treatment biopsy tissue was retrieved for immunohistochemical staining against two endothelial cell markers (CD31 and CD34) and also against VEGF. Angiogenesis was assessed by determining the intratumoural microvessel density (MVD) and the degree of VEGF expression in these specimens. This was correlated with patient outcome in terms of local recurrence, metastasis and death. Histological results were also compared to a group of patients less than 40 years of age.

**Results:**

Of the 11 patients, 9 were male and 2 were female and the mean age was 58 years (range, 42–85). In 7 patients, osteosarcoma arose secondarily from Paget's disease of the bone. The most common site involved was the humerus (7) followed by the femur (2) then pelvis (1) and ulna (1). At the time of diagnosis, 4 patients had metastatic disease. Preoperative chemotherapy was given to 4 patients, with a good response in 3 patients. Six patients underwent limb-sparing surgery, 4 had amputations and 1 was treated with radiotherapy alone. The mean follow up time was 31.5 months (range, 8–81). At this time, 4 patients (36%) had developed lung metastases and 5 patients (46%) had died. Overall survival was 54.5%. Intratumoural MVD was higher in patients over 40 years, although not statistically significant (*p *= 0.111, CD31; *p *= 0.134, CD34). VEGF was uniformly expressed in all sections, however no relationship was found between the degree of expression and patient age.

**Conclusion:**

The prognosis for older patients with osteosarcoma is generally poor. Initial presentation is commonly associated with metastatic disease and neoadjuvant chemotherapy is often avoided because of its side effects. Increased intratumoural vascularity may contribute to the poorer prognosis in these patients, however further studies are needed.

## Introduction

Osteosarcoma is the commonest primary tumour that arises from bone and is the second highest cause of cancer-related death in the paediatric age group. It most commonly afflicts young people in the second and early third decades of life, with over 60% of diagnoses being made between the ages of 10–20 years. However, when osteosarcoma occurs in patients older than the age of 40 years, it is usually in the setting of a pre-existing condition such as Paget's disease or prior irradiated bone and occasionally in osteogenesis imperfecta [1,2]. The prognosis in older patients is notoriously poorer compared to children and adolescents, and several studies have shown that prognosis worsens with age among adults, but is age-independent in children [3]. This is largely attributable to an inability to tolerate high dose chemotherapy, and a tendency towards non-extremity tumour sites [4]. Although the clinical, histopathologic features and prognostic indicators are well defined for young patients, much less is known about affected adults.

Modern treatment of osteosarcoma aims at effectively inhibiting tumour growth, therefore permitting safe tumour resection and subsequent reconstruction of the limb, and prevention of metastatic disease. To achieve this, aggressive and intense cure-oriented combined modality chemotherapy has been developed over the last 2–3 decades. The most commonly used regime comprises of doxorubicin, cisplatin, ifosfamide and high-dose methotrexate. Although there has been a significant improvement in long-term outcome in these patients, 25–50% of patients subsequently develop metastatic disease, and this remains the major cause of death [5]. Furthermore, this form of treatment is often associated with significant morbidity and therefore only justified in younger patients.

In recent times, much research focus has been directed at the role of angiogenesis in tumour development, growth, invasion and metastasis. Angiogenesis, which is most commonly assessed by intratumoural microvessel density (MVD), is the formation of new blood vessels from pre-existing ones, and has shown to correlate with clinicopathological factors and patient prognosis in a variety of tumours including breast, prostate and gastric carcinomas [6-9]. Moreover, it is becoming increasingly clear that tumour angiogenesis is the result of an imbalance between pro-angiogenic and ant-angiogenic factors, and induction of the "angiogenic switch" leads to a threshold change in the favour of pro-angiogenesis [10]. While angiogenic inhibitors have been used increasingly in a number of clinical trials for the treatment of advanced cancers, their potential role as an adjuvant to chemotherapy in osteosarcoma, a hypervascular tumour, is still unclear. Few studies have been performed, reporting varying results and conflicting conclusions. While Mikulic *et al *(2004) showed poorer prognosis in osteosarcoma patients with increased MVD, Matadakis *et al *(2001) failed to demonstrate any association with MVD and outcome [11,12]. On the other hand, Kreuter *et al *(2004) reported that increased angiogenesis is a prognostic indicator for higher survival and favourable response rates to chemotherapy [13].

It is in theory that by targeting tumour endothelial cells or inhibiting certain angiogenic factors, such as VEGF, this may be an effective way of controlling tumour growth with increased specificity and less systemic toxicity. Hence, the purpose of this study was to describe our institution's experience with the management of osteosarcoma in patients over the age of 40 years and also to determine the role of angiogenesis, as a prognostic factor, in these patients.

## Materials and methods

### Patients and tissue specimens

The Bone and Soft Tissue Tumour Service at St. Vincent's Hospital and Peter MacCallum Cancer Institute, Melbourne is an adult tertiary referral centre for the management of musculoskeletal tumour patients in Victoria and Tasmania, Australia and services a population of approximately 4 million people. A retrospective analysis was performed identifying all patients with osteosarcoma that were treated at our institution by the senior author (PFMC) between 1996 and 2004. In order to be eligible for this study, patients had to have presented with newly diagnosed osteosarcoma (either primary or secondary) and had not received neoadjuvant chemotherapy or radiotherapy prior to diagnostic biopsy.

During the study period of 8 years, 46 patients were diagnosed with osteosarcoma at the authors' institution, of which, 11 were over the age of 40 years (24%). An analysis of those patient over the age of 40 years was performed and the following variables were evaluated: age, sex, presentation, clinical course and subsequent treatment, tumour histology, site, response to chemotherapy, the development of metastases and long term survival. Tumours were classified according to predominant histologic features.

In order to evaluate the degree of tumour angiogenesis in this group, archival paraffin-embedded tissue of the pre-treatment diagnostic biopsies were retrieved from our institution's pathology department. Of the 11 patients, there were only 8 cases where sufficient archival paraffin-embedded tissue were available for histological and immunohistochemical analysis. The degree of angiogenesis was then compared to a group of 17 patients with osteosarcoma that were below the age of 40 years.

### Immunohistochemical staining for CD 31, CD34 and VEGF

Archival formalin-fixed paraffin-embedded blocks of the diagnostic core biopsy tissue were retrieved and immunohistochemical staining was performed on 5 μm serial sections. In order to visualise the microvessels, immunostaining was performed using 2 well-known endothelial cell markers, CD31 and CD34. A monoclonal mouse-anti-human antibody to CD31 (DakoCytomation, Glostrup, Denmark) at 1:20 dilution and a monoclonal mouse-anti-human antibody to CD34 (DakoCytomation, Glostrup, Denmark) at 1:25 dilution were used. Immunolocalisation of VEGF was performed using a rabbit polyclonal IgG antibody at 1:100 dilution (Santa Cruz Biotechnology, Santa Cruz, CA). Standard indirect immunohistochemisty was performed using the avidin-biotin-peroxidase complex technique, with minor modifications. Briefly, after deparaffinizing the sections in *Solv21 *(United Biosciences, QLD, Australia) and rehydrating the tissue sequentially through ethanol, antigen retrieval was performed by microwave heating with 10 mM Tris, 1 mM EDTA (pH 9.0) buffer solution for 12 minutes. Sections were treated for 30 minutes with 10% hydrogen peroxide in dH_2_O to block endogenous peroxidase activity. Subsequently, the tissue was immersed in 10% normal rabbit serum for 30 minutes, and then incubated with the primary antibody overnight at 4°C. After two washes in 1× PBS, a biotinylated rabbit-anti-mouse secondary antibody was applied for the CD31 and CD34 stained sections and a biotinylated goat-anti-rabbit secondary antibody (DakoCytomation, Glostrup, Denmark) was applied for the VEGF stained sections (1:300 dilution for 30 minutes). Following this, the slides were washed again in 1× PBS and then treated with peroxidase-conjugated streptavidin for 20 minutes, and the specific antibody binding was visualised using 3,3'-diaminobenzidine tetrahydrochloride (DAB). Specificity of immunoreactivity was confirmed by substitution of the primary antibody with PBS, and in all negative controls, no staining was observed. Light counterstaining with Harris haematoxylin was performed.

### Assessment of intra-tumoural microvessel density (MVD)

The degree of intratumoural angiogenesis was determined by calculating the microvessel density (MVD). Immunohistochemical assessment was performed using light microscopy and the MVD was assessed according to the International Consensus Report, by two independent investigators, who were blinded to the diagnosis, clinical course, management and outcome of the patients [14]. Briefly, the entire section was systematically scanned at 100× magnification and the areas of most intense vascularisation, ie. greatest number of CD31 or CD34-antigen positive cells, was classified as a "hot spot". In order to be defined as a "hot spot", the density of antigen-positive cells and cell clusters need to greater relative to adjacent areas. A positive vessel is a single endothelial cell, endothelial cell cluster, or microvessel that is clearly separated from adjacent microvessels [7]. The magnification was changed to 200× then 400×, and the slide was repositioned over the area of the "hot spot" with the most vessels per field. For each section, the 3 "hottest" spots were assessed and the mean count of all independent readings is calculated and the mean count is defined as the mean count of microvessels per 400× field area (0.26 mm^2^). The MVD was assessed using CD31 and CD34 counts separately.

### Assessment of VEGF expression

Semiquantitative assessment of VEGF expression was made at 200× magnification, based on the overall intensity of membranous and cytoplasmic staining within the tumour cells and the percentage of cells stained. Four grades were given – negative, low (+), moderate (++), and strong (+++). Variations in grading between the two observers were identified and the cases were individually discussed and a final consensus was made.

### Statistical analysis

Results were analysed using MedCalc for Windows v8.1 (Medcalc Software, Belgium). Non-parametric tests were used to allow for data that was not normally distributed. Associations between age and MVD or the degree of VEGF expression were analysed using the Mann-Whitney U test. Correlation between MVD or VEGF expression and patient age was determined using Spearman's Rank co-efficient. A *p*-value ≤ 0.05 was considered significant.

## Results

### Patient and tumour characteristics

Eleven patients above the age of 40 years with osteosarcoma were treated at our institution between 1996 and 2004 (Table 1). The mean age was 58.3 years (range, 42–85 years). Nine patients were male and 2 were female. At the time of presentation, 7 patients had apparent localised disease and 4 had evidence of metastatic disease; 2 had lung metastases, 1 had a subcutaneous metastasis to the calf and 1 had a bony metastasis to the tibia. Osteosarcoma arose secondarily to Paget's disease of the bone in 7 patients. Seven of the tumours were located in the humerus, 2 in the femur, 1 in the proximal ulna, and 1 in the pelvis. No patients presented with pathological fracture.

**Table 1:** Patient and tumour characteristics

**Features**	**No.**
Gender	
Male	9 (81.8%)
Female	2 (18.2%)
Median Age (range)	58.3 (42–85)
Tumour site	
Humerus	7 (63.6%)
Femur	2 (18.2%)
Pelvis	1 (9.1%)
Ulna (proximal)	1 (9.1%)
Primary or Secondary osteosarcoma	
Primary	4 (36.4%)
Secondary – Paget's Disease	7 (63.6%)
Primary metastases	
Absent	7 (63.6%)
Present	4 (36.4%)
Histological subtype	
Osteoblastic	11 (100%)

In terms of histologic subtype, all 11 osteosarcoma were classified as high-grade osteoblastic lesions. Estimates of tumour volume were available in 8 of the 10 patients who subsequently had surgical resection. Tumour volume was calculated as length × width × depth × 0.52 cm^3^, and the mean value was 338.3 cm^3 ^(range, 55.9–709.3 cm^3^).

### Treatment and clinical outcomes

**Figure 1:**
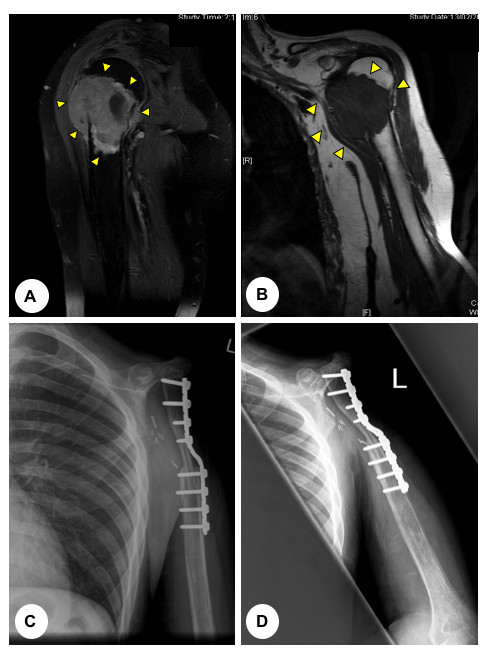
**(A) **T2-weighted and **(B) **T1-weighted coronal magnetic resonance images (MRI) of a 43 year old man presenting with osteosarcoma involving the proximal humerus (arrows). **(C) **Antero-posterior and **(D) **lateral post-operative plain radiographs demonstrating the surgical management, which comprised of a proximal humeral resection and a claviculo-prohumeral reconstruction of the shoulder.

**Figure 2:**
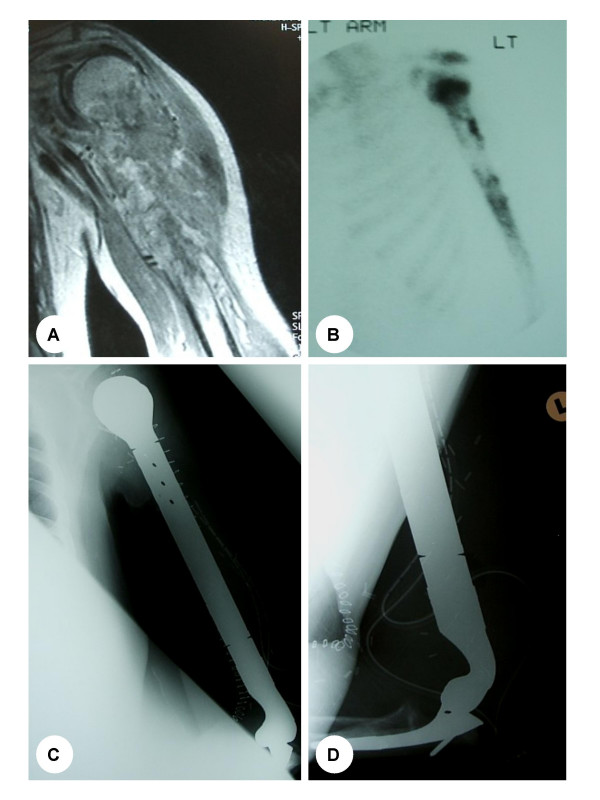
45 year old man with osteosarcoma involving his humerus. **(A) **Coronal T1-weighted MRI demonstrating the tumour involving the proximal half of the humerus. **(B) **Bone scan demonstrates increased uptake in the area of the tumour on the delayed static image. **(C) **and **(D) **Subsequent operative management comprised of a total humeral resection and endoprosthetic reconstruction.

**Figure 3:**
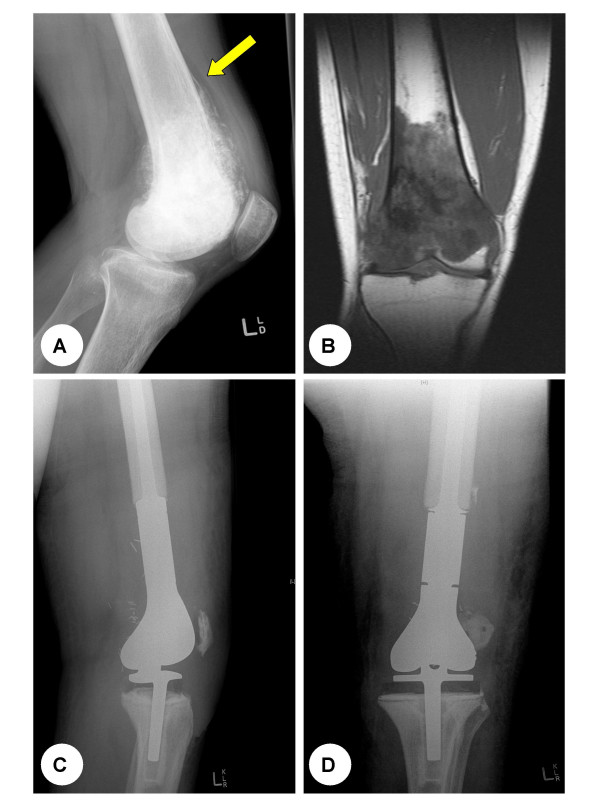
**(A) **Plain lateral radiograph showing the characteristic ill-defined lesion with both lytic and osteoblastic features and evidence of periosteal elevation (Codman's triangle) (arrow). **(B) **T1-weighted magnetic resonance image showing the intramedullary extension of the tumour, with cortical destruction and extension into the soft tissue. **(C) **Lateral and **(D) **anteroposterior plain radiographs post distal femoral resection and reconstruction of the knee joint with a GMRS (Global modular replacement system, Stryker Howedica) megaprosthesis.

**Figure 4:**
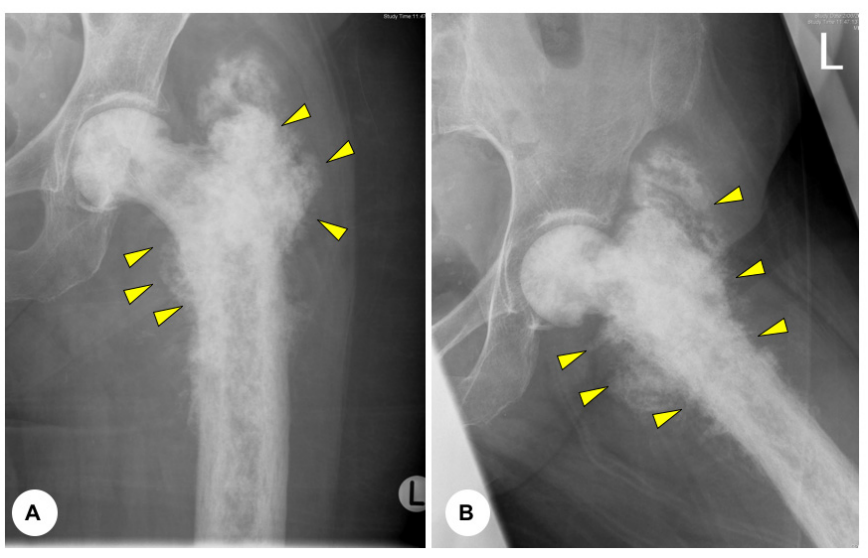
**(A) **and **(B) **Anteroposterior and lateral plain radiographs displaying typical pagetoid changes to the proximal femur with evidence of tumour extending beyond the cortex into the soft tissue (arrows). This 85 year old lady was managed conservative with radiotherapy alone.

Ten of the 11 patients underwent surgical resection of the tumours. Of the 10 patients, 4 had limb amputations (two above elbow, one above knee and one forequarter amputation) and 6 had limb-sparing surgery. For the 4 humeral lesions, reconstructive procedures comprised of 1 resection of the proximal humerus and claviculo-prohumeral reconstruction, 1 allograft replacement and arthrodesis of the proximal humerus, 1 proximal humeral replacement and 1 total humeral replacement (Figures 1, 2). The pelvic osteosarcoma was treated with an internal hemipelvectomy and reconstruction with a saddle prosthesis arthroplasty (Link, Exactech, Florida, USA) to restore a mobile hip joint. One patient had a distal femoral resection and reconstruction of the knee with a Global Modular Replacement System (GMRS, Stryker Howmedica) (Figure 3). One patient received only external beam radiotherapy to the tumour as a form of local palliative control (Figure 4). Adequate margins were achieved in all cases at the time of surgery.

Four patients received both neoadjuvant and adjuvant chemotherapy. The treatment regimes comprised of a combination of standard agents and were administered according to current protocols. These included doxorubicin (n = 4), cisplatin (n = 3), Ifosfamide (n = 1) and high dose methotrexate (n = 1). In terms of histological response, 3 (75%) had a good response (≥ 90% necrosis), and 1 (25%) had a poor response (<90% necrosis). One patient received adjuvant chemotherapy alone and one had a combination of adjuvant chemotherapy and radiotherapy to the lesion. In all cases, the chemotherapy was relatively well tolerated, with no notable toxicity-related complication.

The mean follow up period was 31.5 months (range, 8–81 months). At the time of latest review, 5 patients (46%) had died of their disease. Four patients were continuously disease-free and 2 patients were disease-free after pulmonary metastasectomy. The overall survival was 54.5%. During the time of the study, no patients had developed a local recurrence. Of the 7 patients that were metastasis-free at initial diagnosis, 4 (57%) went on to develop metachronous lesions in the lung at a mean time of 13 months (range, 1–24 months). The management in 3 patients comprised of pulmonary metastasectomy. For the 4 patients that received neo/adjuvant chemotherapy, only 2 (50%) were continuously disease-free at the time of latest follow up (mean, 36 months).

### Assessment of angiogenesis and its correlation with patient age

Intratumoural MVD and the level of VEGF expression was compared between two groups: patients above and below the age of 40 years. The mean age of the patients over 40 years (n = 8) was 56.1 years (range, 42–85 years), while those less than 40 years (n = 17) was 21.6 years (range, 13–36 years).

**Figure 5:**
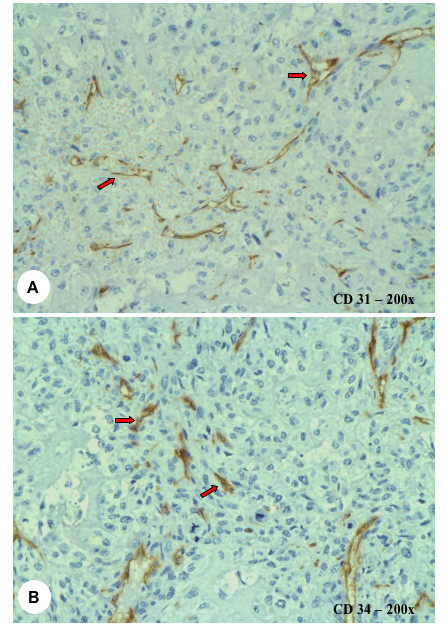
**(A) **CD31 and **(B) **CD34 immunostaining demonstrating the intratumoural microvessels (arrows). Magnification 200×.

For the patients older than 40 years, the median MVD from CD31 immunostaining was 37 microvessels per 0.26 mm^2 ^(interquartile range of 23 to 49). From CD34 expression, the median MVD was 52 microvessels per 0.26mm^2 ^(interquartile range of 32 to 60). In the group of patients less than 40 years, the median MVD from CD31 immunostaining was 22 microvessels per 0.26 mm^2 ^(interquartile range of 18 to 30). The median MVD from CD34 expression was 29 microvessels per 0.26 mm^2 ^(interquartile range of 23 to 46) (Figure 5). From these results, for both CD31 and CD34, the median MVD was higher in the older group of patients. However, although this did not demonstrate any statistically significant difference (*p *= 0.111, CD31; *p *= 0.134, CD34), there is evidence that a trend may exist to suggest that patients over the age of 40 years have a higher MVD compared to those less than 40 years.

**Figure 6:**
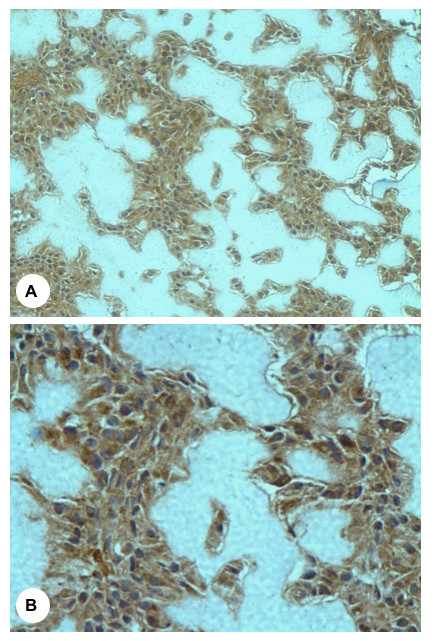
Photomicrographs demonstrating immunohistochemical staining of osteosarcoma tissue for VEGF. Panels **(A) **and **(B) **are a 200× and 400×, respectively.

**Figure 7:**
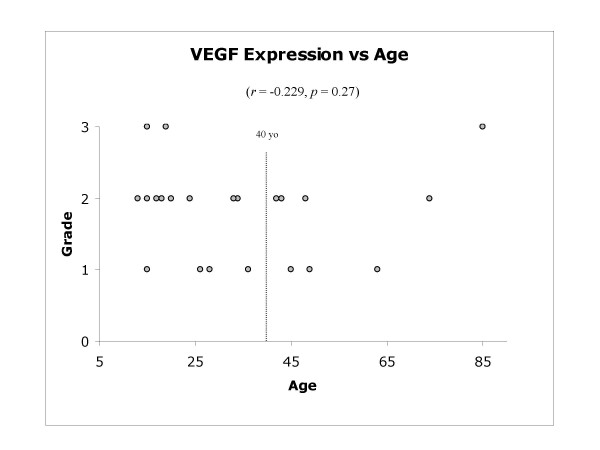
**VEGF staining intensity according to patient age**. No statistical correlation was found between the degree of VEGF staining and increasing patient age. The dotted line delineates patients above and below the age of 40 years. No statistical difference was noted between the two groups.

In terms of VEGF expression, all of the 25 tumour specimens showed positive cytoplasmic VEGF protein expression in the tumour cells (Figure 6). Superficial examination of the specimens at 100× magnification revealed apparent localised variation in the expression of VEGF protein, with areas of negative expression interspersed throughout the sections. In the great majority of cases, this corresponded to areas of increased microvessels density ("hot spots") as determined by CD31 and CD34 immunostaining. However, there was no significant difference observed between the two groups (*p *= 0.66). Furthermore, no correlation was seen between the degree of VEGF expression and increasing patient age (*r *= -0.229, *p *= 0.27) (Figure 7). No significant correlation was demonstrated in the MVD and VEGF expression between patients with primary and secondary (Paget's disease) osteosarcoma. Moreover, in patients who had received neo/adjuvant chemotherapy, no statistical significance was noted between VEGF staining, patient gender, the development of recurrence, metastasis or death.

## Discussion

The incidence of osteosarcoma has a well-recognised double peak. Although, the majority of tumours arise in patients in their adolescence, there is a significant second peak in the later years of life, with approximately 13% of patients being over the age of 40 years [15]. Most studies have concluded that patients over the age of 40 years fare considerably worse compared to their younger counterparts, which may be attributable to their potential inability to tolerate aggressive chemotherapeutic agents and also a tendency towards non-extremity tumour sites [16]. The prognostic significance, however of age in osteosarcoma is generally difficult to assess, as in most clinical trials and multivariate analyses, older patients are excluded. Although Bacci *et al *reported that the overall survival in 29 patients over the age of 40 years was not worse than patients under 40 years, Naka *et al *showed a dismal 18% 5-year survival rate for 12 patients and Carsi *et al *published a 42% overall survival rate [3,16,19]. This disparity in outcome is in the context that since the advent of multi-modal high dose chemotherapy as an adjunct to the surgical management of osteosarcoma, the long-term survival for osteosarcoma, across all ages, has risen to approximately 70% [4]. In this present study, we attempted to determine by immunohistochemistry, whether increased microvessel density or the expression of pro-angiogenic factors, such as VEGF, in older patients might be an attributable factor in the poorer prognosis in this age group.

The concept that malignant tumour development, growth, invasion and metastasis depends on angiogenesis is now a widely recognised and accepted theory. Several clinical studies on various types of malignant tumours have supported this demonstrating a positive correlation between intratumoural microvessel density and certain clinicopathological factors and patient prognosis [6]. Tumour angiogenesis is a result of an imbalance between pro-angiogenic and anti-angiogenic factors [20]. Folkman postulated that dormant avascular tumour nodules could only grow and develop if they became vascularized. Therefore, the induction of the "angiogenic switch" on tumours, to develop an angiogenic phenotype is key process in malignant invasion and metastasis [21,22]. Thus, there is a threshold change in the balance between stimulatory and inhibitory influences, in favour of pro-angiogenesis. In recent times, much research focus has been directed towards investigating whether interruption of this process could theoretically halt the progression of tumours that are dependent on angiogenesis for further growth. This therapeutic potential could augment the effects of chemotherapy and radiotherapy by limiting the tumour to a dormant state of low metastatic potential [23].

VEGF is one of the most potent known stimulators of angiogenesis and acts by activating endothelial cells via interaction with its receptors, Flk-1 and Flt-1, which are selectively expressed in the endothelium [24]. The important role of VEGF in tumour metastasis has been demonstrated by positive correlation between VEGF expression and the development of metastatic disease and overall prognosis in gastric, colorectal and oesophageal carcinoma [25-27]. This association is further echoed in osteosarcoma with positive VEGF expression correlating with increased local microvessel density, development of pulmonary metastasis and poor outcome [28].

In our study, we did not observe any significant correlation between the grade of VEGF immunostaining and patient age. Furthermore, the degree VEGF expression did not seem to be associated with other clinicopathological parameters such as the development of local recurrence, metastasis and death. In our observation of 25 cases, positive VEGF expression was demonstrated in all tumour "hot spots", which may be an expected finding considering VEGF is a potent pro-angiogenic factor. However, the premise of assessing the degree of VEGF protein expression was to determine whether there was variation in angiogenic factor regulation between different tumours, and in turn, whether this had any relationship to tumour progression. Therefore, although the degree of expression may not provide prognostic significance, it is likely to be of key functional importance, as it is uniformly expressed in all tumour samples.

## Conclusion

In this present study, the aims were two-fold; firstly to describe our institution's experience with the management of osteosarcoma in older patients, and secondly to determine whether there was any correlation between the intratumoural microvessel density or the degree of pro-angiogenic factor expression (VEGF) and patient age. The results of this study have demonstrated that the intratumoural microvessel density was higher in patients over the age of 40 years, and although this was not statistically significant, there may be suggestion that a trend may exist. Furthermore, VEGF was uniformly expressed in all osteosarcoma specimens, however no significant relationship was found between the degree of VEGF staining and patient age. We do appreciate that this is a small series, however we have observed that the overall survival is poorer in older patients, and this is consistent with current literature. This may be attributed to increased intratumoural vascularity, however further studies need to be performed. If this is the case, there may be a potential role for anti-angiogenic therapy, as an augment to chemotherapy, for these more elderly patients with osteosarcoma.

## Competing interests

The author(s) declare that they have no competing interests.

## Authors' contributions

EE was the principle investigator and drafted the manuscript. JO was a co-investigator and assisted with the analysis of data and the preparation of manuscript. YK assisted in assessment of data and assisted with the preparation of manuscript. PC was the supervising author and was the surgeon involved in the management of the patients studied. All authors read and approved the final manuscript.
